# Evaluation of the effect of intraperitoneal etanercept administration on oxidative stress and inflammation indicators in the kidney and blood of experimental sepsis-induced rats

**DOI:** 10.1590/0037-8682-0016-2020

**Published:** 2020-04-27

**Authors:** Emre Aydın, Yaşar Yıldırım, Fatma Yılmaz Aydın, Mehmet Veysi Bahadır, İbrahim Kaplan, Berfin Kadiroğlu, Muzaffer Aydın Ketani, Zülfükar Yılmaz, Ali Kemal Kadiroğlu, Mehmet Emin Yılmaz

**Affiliations:** 1 University of Dicle, School of Medicine, Department of Nephrology, Diyarbakır, Turkey.; 2 University of Dicle, School of Medicine, Department of Internal Medicine, Diyarbakır, Turkey.; 3 University of Dicle, School of Medicine, Department of General Surgery, Diyarbakır, Turkey.; 4 University of Dicle, School of Medicine, Department of Biochemistry, Diyarbakır, Turkey.; 5 University of Dicle, School of Veterinary Medicine, Department of Virology, Diyarbakır, Turkey.; 6 University of Dicle, School of Veterinary Medicine, Department of Histology and Embryology, Diyarbakır, Turkey.

**Keywords:** Etanercept, Oxidative stress, Triggering receptor expressed on myeloid cells, Sepsis, TNF-alpha

## Abstract

**INTRODUCTION:**

Sepsis is an important cause of mortality and morbidity, and inflammatory response and oxidative stress play major roles underlying its pathophysiology. Here, we evaluated the effect of intraperitoneal etanercept administration on oxidative stress and inflammation indicators in the kidney and blood of experimental sepsis-induced rats.

**METHODS:**

Twenty-eight adult Sprague Dawley rats were classified into Control (Group 1), Sepsis (Group 2), Sepsis+Cefazolin (Group 3), and Sepsis+Cefazolin+Etanercept (Group 4) groups. Kidney tissue and serum samples were obtained for biochemical and histopathological investigations and examined for the C reactive protein (CRP), tumor necrosis factor-alpha (TNF-α), triggering receptor expressed on myeloid cells (TREM), and malondialdehyde (MDA) levels.

**RESULTS:**

The levels of TNF-α, TREM, and MDA in serum and kidney samples were significantly higher in rats from sepsis group than in rats from control group (p < 0.05). Group 3 showed a significant reduction in serum levels of TNF-α, CRP, and TREM as compared with Group 2 (p < 0.05). Serum TNF-α, CRP, TREM, and MDA levels and kidney TNF-α and TREM levels were significantly lower in Group 4 than in Group 2 (p < 0.05). Serum TNF-α and TREM levels in Group 4 were significantly lower than those in Group 3, and histopathological scores were significantly lower in Group 3 and Group 4 than in Group 2 (p < 0.05). Histopathological scores of Group 4 were significantly lower than those of Group 3 (p < 0.05).

**CONCLUSIONS:**

Etanercept, a TNF-α inhibitor, may ameliorate sepsis-induced oxidative stress, inflammation, and histopathological damage.

## INTRODUCTION

Sepsis is a life-threatening organ dysfunction that occurs in response to an infection. Sepsis is also one of the most common causes of death worldwide. Early diagnosis has been hypothesized to significantly reduce the morbidity and mortality associated with sepsis^1^.

Inflammatory response plays an important role in the pathophysiology of sepsis^2^. Lipopolysaccharides in the cell wall of gram-negative bacteria trigger the release of many pro-inflammatory cytokines such as interleukin (IL)-1, IL-6, and the tumor necrosis factor-alpha (TNF-α) by affecting monocytes and macrophages^3^. To limit the tissue damage, the overexpressed pro-inflammatory cytokines may induce a pathological response. In particular, TNF-α plays a major role in the development of sepsis-induced organ dysfunction. Controlling the pathological effects of pro-inflammatory cytokine overproduction may be an important strategy in the treatment of sepsis. Therefore, many in vitro and in vivo studies have been performed, and various therapeutic agents have been studied. One of these agents is etanercept, a fusion protein formed by recombinant DNA. Etanercept binds to TNF-α and inhibits its binding with TNF-α receptors on the cell surface. Thus, the efficacy of TNF-α is reduced, thereby suppressing the inflammatory response. This phenomenon may be particularly important to prevent organ dysfunction in sepsis^4^ ^-^ ^6^.

Infection is often followed by a natural immune response. The early step in the activation of the innate immune response is mediated by the interaction of macrophages, leukocytes, and the Toll-like receptors (TLRs) on endothelial cell surfaces with various microbial products. The triggering receptors expressed on myeloid cells (TREM) is a member of the immunoglobulin superfamily found on surfaces of neutrophils, monocytes, and macrophages and mainly interacts with TLR ligands^7^. TREM activation leads to the production of pro-inflammatory cytokines and chemokines, upregulation in the expression of stimulatory molecules, suppression in IL-10 levels, induction of neutrophil degranulation, and enhancement in phagocytic activities^8^. In many studies, TREM level was found to be increased on surfaces of neutrophils and monocytes in circulation upon exposure to infectious agents^9^. In addition, TREM was described as a diagnostic biomarker in both early and late-onset neonatal sepsis^10^, and proposed to serve as an immunohistochemical marker for the diagnosis of sepsis^11^.

Oxidative stress is as important as an inflammatory response in the pathophysiology of sepsis. Many studies with human and animal sepsis models have confirmed the presence of severe oxidative stress in subjects with sepsis. The disturbance in the oxidant-antioxidant balance towards an oxidant dominance during inflammation results in the induction of oxidative stress. The contact of free oxygen radicals with fatty acids leads to lipid peroxidation and the subsequent release of several reactive aldehydes, including malondialdehyde (MDA). The consequences include oxidative modification, apoptosis, and tissue damage at the cellular level, subsequently causing an organ dysfunction^12^ ^-^ ^16^. Oxidative stress has been hypothesized to be a therapeutic target because it serves as a powerful mechanism underlying the progression of organ dysfunction^17^. MDA is an important oxidative stress parameter, as it is released from damaged cells in response to lipid peroxidation. Therefore, MDA has been used to demonstrate oxidative stress in sepsis models^16^ ^-^ ^18^.

Sepsis leads to significant organ dysfunctions such as an acute kidney injury, central nervous system damage, cardiovascular dysfunction, lung injury, hepatic dysfunction, and ileus. In particular, an acute renal injury is a common complication of sepsis and an important mortality-related factor^19^ ^-^ ^20^. Therefore, it is crucial to diagnose sepsis at an early stage and initiate an appropriate treatment. In this study, we explored the effect of etanercept on the inhibition of the pro-inflammatory cytokines TNF-α and TREM and monitored the oxidative stress response using MDA. We investigated whether etanercept ameliorated the oxidative stress, inflammatory mediator expression, and histopathological damage in sepsis-induced rats.

## METHODS

This study was performed at the Dicle University Health Sciences Application and Research Center (DUSAM) after obtaining approval from the ethics committee (project number 15.TF.015). A total of 28 adult Sprague Dawley rats weighing 200 g-250 g were used. These rats were fed with the same diet and water for 1 week.

The rats were divided into four study groups with seven rats per group as follows:

Group 1 (Control group): Group without test agent administration.

Group 2 (Sepsis group): Group with sepsis induced on the peritonitis floor by an intraperitoneal injection of a 1.5-mL (10^7^ CFU/mL) *Escherichia coli* suspension. Blood and kidney tissue samples were obtained after laparotomy on day 7.

Group 3 (Sepsis + cefazolin): This group was daily administered with an intraperitoneal injection of cefazolin (50 mL/kg) starting from 1 h after the injection of a 1.5-mL (10^7^ CFU/mL) *E. coli* suspension to induce an experimental sepsis. Blood and kidney tissue samples were obtained after laparotomy on day 7.

Group 4 (Sepsis + cefazolin + etanercept): This group was subjected to a daily intraperitoneal cefazolin (50 mL/kg) injection starting from 1 h after the injection of a 1.5-mL (10^7^ CFU/mL) *E. coli* suspension to induce an experimental sepsis. Further, intraperitoneal etanercept (each dose, 8 mg/kg) injections were administered 1 h and 4 h after the injection of the*E. coli* suspension. Blood and kidney tissue samples were obtained after laparotomy on day 7. The experiment was terminated 7 days after etanercept treatment. 

Ketamine hydrochloride (70 mg/kg) was intramuscularly (IM) injected into rats as an anesthetic prior to an operation. The anesthetized rats were fixed in the supine position. After application of povidone iodine, a midline incision was made on the anterior abdominal wall of rats from all groups for histopathological and biochemical investigation. Kidney tissue samples were fixed with 4% formalin and embedded in paraffin. Five-micron sections were obtained and stained with hematoxylin-eosin (H&E) and the Masson’s trichrome. All samples were examined. Blood samples (5 mL) were obtained from the heart by sternotomy for biochemical examinations. This method was also used to kill the experimental animals.

### Biochemical analysis

The blood samples obtained from rats were centrifuged at 3000 g for 8 min at 4C, and the supernatants were used for analysis. Tissue samples were stored in a refrigerator at −20°C until being homogenized using a homogenizer. Tissue and blood samples were evaluated for the level of the C-reactive protein (CRP), TNF-α, TREM, and MDA at the Central Laboratory of Dicle University Hospital.

Serum levels of TNF-α, CRP, and TREM were assessed using the Sunred Rat kits, which are solid-phase sandwich enzyme-linked immunosorbent assays (ELISAs). MDA level was spectrophotometrically measured using a Shimadzu UV-1201 spectrophotometer (Shimadzu Co., Kyoto, Japan). This method was based on the color product of the reaction between thiobarbituric acid and MDA. The concentration of the thiobarbituric acid-reactive substances was calculated from the absorbance coefficient of the malondialdehyde-thiobarbituric acid complex and indicated as micromole per liter. As an MDA standard, bis (dimethyl-acetal)-thiobarbituric acid complex was used.

### Histopathological analysis

The kidney tissue samples from rats were cut into 4-5-μm paraffin sections using a rotary microtome. These sections were stained with H&E and investigated using a Nikon Eclipse 400 digital camera (Nikon DSRi). Histopathological changes were scored between 0 and 3 depending on the level of tubular necrosis, tubular atrophy, regenerative atypia, hydropic degeneration, interstitial fibrosis, and brushy margin loss as follows: 0, none; 1, mild (focal < 25%); 2, moderate (multifocal 25%-50%); 3, severe (diffuse > 50%)^21^ (Table 1).

**TABLE 1:** Histopathological scoring system.

Score	0	1	2	3
**Tubular necrosis**	Absent	25%	25-50%	> 50%
**Tubular atrophy**	Absent	Focal	Multifocal	Diffuse
**Regenerative atypia**	Absent	Focal	Multifocal	Diffuse
**Hydropic degeneration**	Absent	Focal	Multifocal	Diffuse
**Interstitial fibrosis**	Absent	Focal	Multifocal	Diffuse
**Loss of brush border**	Absent	Focal	Multifocal	Diffuse

### Statistical analyses

The Statistical Package for Social Sciences (SPSS), Version 24.0, for Windows was used to perform data analyses. All data have been reported as mean ± standard deviation. One-way analysis of variance (ANOVA) with a post-hoc Bonferroni correction was conducted for comparison between multiple groups. A value of p < 0.05 was considered statistically significant.

## RESULTS

In comparison with the animals from the control group, those that underwent sepsis had significantly higher levels of serum CRP, TNF-α, TREM, and MDA (p < 0.05). Pretreatment of rats with cefazolin resulted in a significant reduction in the serum levels of TNF-α, CRP, and TREM as compared with the rats from the sepsis group (p < 0.05). Further, the rats treated with cefazolin and etanercept demonstrated significantly reduced levels of serum TNF-α, CRP, TREM, and MDA as compared with those from the sepsis group (p < 0.05). The serum levels of TNF-α and TREM were significantly lower in the rats from the sepsis + cefazolin + etanercept group than in those from the sepsis + cefazolin group. 

Analysis of the kidney tissue samples revealed the significantly higher levels of TNF-α, TREM, and MDA in the sepsis group than those in the control group (p < 0.05). The level of TNF-α in the kidney tissue was found to be significantly higher in the sepsis + cefazolin group than thatin the control group (p < 0.05). However, TREM and TNF-α levels in the kidney tissue were significantly lower in the sepsis + cefazolin + etanercept group than those in the sepsis group (p < 0.05). Table 2 shows levels of oxidative stress and inflammation parameters in the serum and kidney tissue samples of the study groups. 

**TABLE 2:** Comparison of serum and kidney levels of oxidative stress and inflammation indicator parameters.

Parameters	Group 1	Group 2	Group 3	Group 4
	Control	Sepsis	Sepsis + Cefazolin	Sepsis + Cefazolin + Etanercept
	(n = 7)	(n = 7)	(n = 7)	(n = 7)
**Serum**				
CRP (pmol/L)	18.43 ± 6.37	46.50 ± 6.12^a^	26.40 ± 5.30^b^	20.07 ± 5.27^b^
TNF-α (ng/L)	41.74 ± 13.73	149.78 ± 25.02^a^	109.14 ± 8.15^a,b^	45.54 ± 3.7^b,c^
TREM (pg/mL)	93.77 ± 11.55	172.03 ± 19.69^a^	144.12 ± 23.03^a,b^	97.78 ± 14.60^b,c^
MDA (nmol/mL)	1.49 ± 0.54	3.07 ± 0.71^a^	2.33 ± 0.72	1.50 ± 0.41^b^
**Kidney tissue**				
CRP (pmol/L)	23.52 ± 10.29	32.88 ± 19.35	27.26 ± 11.97	21.47 ± 5.41
TNF-α (ng/L)	33.88 ± 10.07	93.07 ± 41.95^a^	78.5 ± 23.84^a^	44.67 ± 13.76^b^
TREM (pg/mL)	54.77± 14.96	146.89 ± 76^a^	116.35 ± 18.62	59.66 ± 25.07^b^
MDA (nmol/mL)	0.66 ± 0.36	1.39 ± 0.63^a^	1.18 ± 0.43	1.01 ± 0.06

**CRP:** C-reactive protein, **TNF-α:** tumor necrosis factor alpha, **TREM:** triggering receptor expressed on myeloid cells, **MDA:** malondialdehyde. ^a^p < 0.05 in comparison with control group. ^b^p < 0.05 in comparison with sepsis group. ^c^p < 0.05 in comparison with sepsis + cefazolin group

Histopathological evaluation of the kidney tissue from different groups showed the following results:

**Control group:** Glomerular and tubular lesions were normal, and no lesions were found in the kidney tissue.

**Sepsis group:** Disturbance in the glomerular structure, dilatation in the Bowman’s capsule, and diffused hydropic degeneration and atrophic structure in the distal and proximal tubules were observed. While no case of interstitial fibrosis was noted, diffused brushy margin loss was evident in the proximal kidney tubules. In addition, desquamation of the tubular epithelium was prominent (score 3).

**Sepsis + cefazolin group:** Although the changes were similar to those observed in group 2, the severity decreased (score 2).

**Sepsis + cefazolin + etanercept group:** The deterioration in the glomerular structure and diffused hydropic degeneration in the distal and proximal tubules decreased. While no interstitial fibrosis was reported, rare brushy edge loss was observed in the proximal kidney tubules. In addition, desquamation of the tubular epithelium was found to be rare (score 1).

**FIGURE 1:**
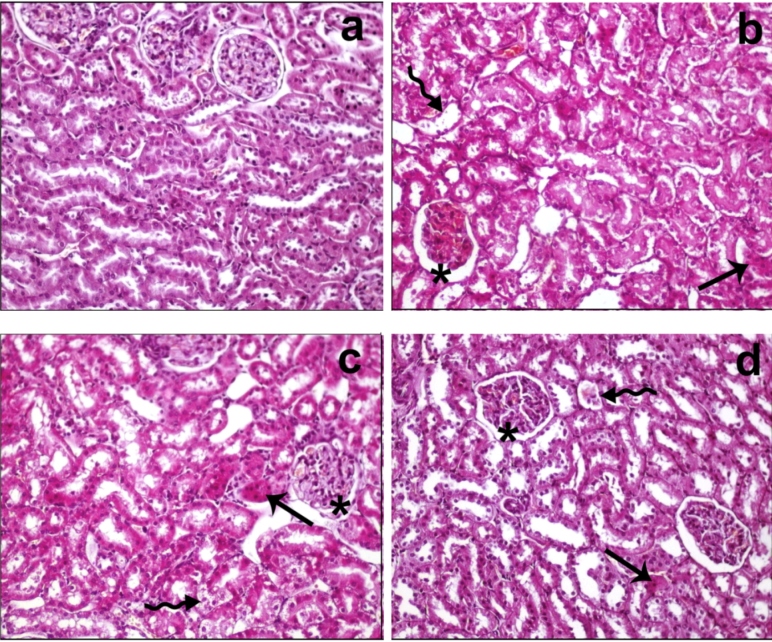
Histopathological findings of the study groups. **a:** In control group, the kidney tissue showed normal morphology, **b:** In sepsis group, hydropic degeneration (arrow), desquamation in tubular epithelium (curved arrow), disturbance in glomerular structures, and dilatation in Bowman’s capsule (*) were observed **c:** In sepsis + cefazolin group, hydropic degeneration (arrow), desquamation in tubular epithelium (curved arrow), and a decrease in dilated Bowman’s capsule (*) were reported **d:** In sepsis + cefazolin + etanercept group, rare hydropic degeneration (arrow), desquamation in tubular epithelium (curved arrow), and dilatation in Bowman’s capsule (*) were visible (staining, H&E; original magnification, ×200).

Histopathological images of the samples from different groups have been presented in Figure 1.

The histopathological score in terms of loss of the brush border, tubular necrosis, and tubular atrophy was significantly lower in sepsis + cefazolin and sepsis + cefazolin + etanercept treatment groups than those in the sepsis group (p < 0.05). Further, the histopathological score for the rats from sepsis + cefazolin + etanercept group was significantly lower than that for the rats from sepsis + cefazolin group (p < 0.05). Histopathological results of kidney tissue samples have been shown in Table 3. 

**TABLE 3:** Histopathological results.

Histopathological findings	Group 1	Group 2	Group 3	Group 4
	Control	Sepsis	Sepsis + cefazolin	Sepsis + cefazolin + etanercept
	(n = 7)	(n = 7)	(n = 7)	(n = 7)
**Tubular necrosis**	0	2.71 ± 0.48^a^	2.14 ± 0.37^a,b^	1 ± 0^a,b,c^
**Tubular atrophy**	0	2.85 ± 0.37^a^	2 ± 0^a,b^	1 ± 0^a,b,c^
**Regenerative atypia**	0	0	0	0
**Hydropic degeneration**	0	3 ± 0	2 ± 0	1 ± 0
**Interstitial fibrosis**	0	0	0	0
**Loss of brush border**	0	2.85 ± 0.37^a^	2.14 ± 0.37^a,b^	0.14 ± 0.37^b,c^

^a^p < 0.05 in comparison with control group. ^b^p < 0.05 in comparison with sepsis group. ^c^p < 0.05 in comparison with sepsis + cefazolin group.

## DISCUSSION

Sepsis is defined as a complex body response to infection. The key mechanisms underlying the pathogenic cascade of sepsis are the immune response, inflammatory response, and oxidative stress. Cellular damage induced by sepsis is accompanied with an increase in oxidative stress, and an inflammatory response often causes organ dysfunctions. The consequences include the multiple organ dysfunction syndrome (MODS), deterioration of the clinical situation, and, eventually, death^22^ ^-^ ^23^.

Our study showed that the serum and kidney levels of TREM and MDA were higher in the rats from the sepsis group than in those from the control group (p < 0.05). Gibot et al. found that patients with sepsis had higher serum TREM levels than those without sepsis and that the TREM level was more sensitive than procalcitonin and CRP^24^. Further, Arizaga-Ballesteros et al. demonstrated the role of TREM as a biomarker in septic shock with a sensitivity of 78% and specificity of 97% ^25^. These studies indicate that the level of TREM increases in sepsis. MDA level has also been used as a parameter to monitor oxidative stress in sepsis. Lorente et al. showed that MDA, an indicator of lipid peroxidation, increased in the serum of adults with sepsis^26^. Sener et al. revealed the upregulated level of MDA in the liver and kidney tissues of mice with sepsis^27^. Andresen et al. also reported similar results, wherein the serum MDA level in severe septic patients was higher than that in healthy controls^28^. The high serum and kidney levels of TREM and MDA in rats from sepsis group observed in our study were consistent with the above-mentioned reports.

Pretreatment of rats from sepsis + cefazolin group with cefazolin led to a significant reduction in the serum levels of CRP, TNF-α, and TREM as compared with rats from sepsis group. Saldir et al. showed that TREM level significantly decreased after 48 h-72 h from the initiation of an antibiotic treatment^29^. In our study, inflammatory parameters significantly decreased following a treatment with cefazolin. 

The main aim of our study was to evaluate the clinical outcomes following administration of etanercept to rats with sepsis. Consistent with the findings after cefazolin administration, the pretreatment of rats with cefazolin and etanercept significantly reduced the serum levels of CRP, TNF-α, TREM, and MDA as well as the kidney levels of TREM and TNF-α. We also observed a significantly lower levels of serum TNF-α and TREM in the rats from sepsis + cefazolin + etanercept group than in those from sepsis + cefazolin group. These results were consistent with those reported in previous studies. Fei et al. showed that the combination treatment with an antibiotic and a TNF inhibitor could significantly reduce staphylococcal septic arthritis and sepsis as compared with an antibiotic treatment alone^30^. Nielsen et al. argued that antibiotic monotherapy was insufficient for the treatment of septic arthritis and that the combination of an antibiotic and an anti-inflammatory agent may be more useful for preventing joint inflammation and bone damage^31^. Ilhan et al. found that the administration of thalidomide and etanercept as therapeutic agents resulted in a significant decrease in the level of TNF-α^32^. Ilhan et al. conducted an experimental study with etanercept in septic rats and found that serum MDA level significantly decreased following an etanercept treatment^33^. In line with these findings, our results showed that etanercept significantly reduced inflammatory and oxidative stress parameters in sepsis. 

We analyzed kidney tissue samples and found a statistically significant difference in the level of TREM between sepsis + cefazolin + etanercept and sepsis groups (p < 0.05). We also reported improvement in the histopathological findings in kidney tissue samples treated with etanercept. Histopathological scores of loss of the brush border, tubular necrosis, and tubular atrophy were significantly lower in sepsis + cefazolin and sepsis + cefazolin + etanercept groups than those in the sepsis group (p < 0.05). Further, the histopathological score of sepsis + cefazolin + etanercept group was significantly lower than that of sepsis + cefazolin group (p < 0.05). These results indicate that the addition of etanercept to antibiotics may ameliorate histopathological changes in the kidney tissue. Fei et al. performed histopathological examination of the kidney tissues and reported lower rates of tubular degeneration in the anti-TNF-treated group than those in the antibiotic alone group, consistent with the results of our study^30^.

In conclusion, we demonstrate that the pretreatment with etanercept, a TNF-α inhibitor, may ameliorate sepsis-induced oxidative stress, inflammation, and kidney damage. More studies are warranted to verify the positive effects of etanercept on antibiotic therapy and oxidative stress and inflammatory parameters in sepsis rats.

## References

[1] Singer M, Deutschman CS, Seymour SW, Shankar-Hari M, Annane D, Bauer M (2016). “The third international consensus definitions for sepsis and septic shock (sepsis-3)”. JAMA.

[2] Ding R, Meng Y, Ma X (2018). The Central Role of the Inflammatory Response in Understanding the Heterogeneity of Sepsis-3. BioMed Res Int.

[3] Lo´pez-Bojo´rquez LN, Dehesa AZ, Reyes-Tera´n G (2004). Molecular Mechanisms, Involved in the Pathogenesis of Septic Shock. Arch Med Res.

[4] Iba T, Miyasho T (2008). Danaparoid sodium attenuates the increase in inflammatory cytokines and preserves organ function in endotoxemic rats. Crit Care.

[5] Ren Y, Xie Y, Jiang G, Fan J, Yeung J, Li W (2008). Apoptotic cells protect mice against lipopolysaccharide-induced shock. J Immunol.

[6] Braun J, McHugh N, Singh A, Wajdula JS, Sato R (2007). Improvement in patient-reported outcomes for patients with ankylosing spondylitis treated with etanercept 50 mg once-weekly and 25 mg twice-weekly. Rheumatology.

[7] Oku R, Oda S, Nakada TA, Sadahiro T, Nakamura M, Hirayama Y (2013). Differential pattern of cell-surface and soluble TREM-1 between sepsis and SIRS. Cytokine.

[8] Arts RJ, Joosten LA, Van der Meer JW, Netea MG (2013). TREM-1: intracellular signaling pathways and interaction with pattern recognition receptors. J Leukoc Biol.

[9] Bouchon A, Dietrich J, Colonna M (2000). Inflammatory Responses Can Be Triggered by TREM-1, a Novel Receptor Expressed on Neutrophils and Monocytes. J Immunol.

[10] Patoulias D, Kalogirou MS, Patoulias I (2018). Triggering Receptor Expressed on Myeloid Cells-1 (TREM-1) and its soluble in the plasma form (sTREM-1) as a diagnostic biomarker in neonatal sepsis. Folıa Med Cracov.

[11] Maiese A, Bolino G, Mastracchio A, Frati P, Fineschi V (2019). An immunohistochemical study of the diagnostic value of TREM-1 as marker for fatal sepsis cases. Biotech Histochem.

[12] Rocha M, Herance R, Rovira S, Hernández-Mijares A, Victor VM (2012). Mitochondrial dysfunction and antioxidant therapy in sepsis. Infect Disord Drug Targets.

[13] Galley HF (2011). Oxidative stress and mitochondrial dysfunction in sepsis. Br J Anaesth.

[14] Galley HF (2010). Bench-to-bedside review: targeting antioxidants to mitochondria in sepsis. Crit Care.

[15] Draper HH, Hadley M (1990). Malondialdehyde determination as index of lipid peroxidation. Methods Enzymol.

[16] Dalle-Donne I, Rossi R, Colombo R, Giustarini D, Milzani A (2006). Biomarkers of oxidative damage in human disease. Clin Chem.

[17] Weiss SL, Deutschman CS (2014). Elevated malondialdehyde levels in sepsis - something to ‘stress’ about?. Crit Care.

[18] Mishra V, Baines M, Wenstone R, Shenkin A (2005). Markers of oxidative damage, antioxidant status and clinical outcome in critically ill patients. Ann Clin Biochem.

[19] Zheng YH, Xiong B, Deng YY, Lai W, Zheng SY, Bian HN (2017). Effects of allogeneic bone marrow mesenchymal stem cells on polarization of peritoneal macrophages in rats with sepsis. Zhonghua Shao Shang ZaZhi.

[20] Vandijck DM, Reynvoet E, Blot SI, Vandecasteele E, Hoste EA (2007). Severe infection, sepsis and acute kidney injury. Acta Clin Belg.

[21] Kudose S, Hoshi M, Jain S, Gaut JP (2018). Renal Histopathologic Findings Associated with Severity of Clinical Acute Kidney Injury. Am J Surg Pathol.

[22] Binkowska AM, Michalak G, Słotwinski R (2015). Current views on the mechanisms of immune responses to trauma and infection. Cent Eur J Immunol.

[23] Arlati S, Storti E, Pradella V, Bucci L, Vitolo A, Pulici M (2007). Decreased fluid volume to reduce organ damage: A new approach to burn shock resuscitation? A preliminary study. Resuscitation.

[24] Gibot S, Kolopp-Sarda MN, Béné MC, Cravoisy A, Levy B, Faure GC (2004). Plasma level of a triggering receptor expressed on myeloid cells-1: its diagnostic accuracy in patients with suspected sepsis. Ann Intern Med.

[25] Arízaga-Ballesteros V, Alcorta-García MR, Lázaro-Martínez LC, Amézquita-Gómez JM, Alanís-Cajero JM, Villela L (2015). Can sTREM-1 predict septic shock & death in late-onset neonatal sepsis? A pilot study. Int J Infect Dis.

[26] Lorente L, Martín MM, Abreu-González P, Domínguez-Rodriguez A, Labarta L, Díaz C (2013). Sustained high serum malondialdehyde levels are associated with severity and mortality in septic patients. Crit Care.

[27] Sener G, Toklu H, Kapucu C, Ercan F, Erkanli G, Kaçmaz A (2005). Melatonin protects against oxidative organ injury in a rat model of sepsis. Surg Today.

[28] Andresen M, Regueira T, Bruhn A, Perez D, Strobel P, Dougnac A (2008). Lipoperoxidation and protein oxidative damage exhibit different kinetics during septic shock. Mediators Inflamm.

[29] Saldir M, Tunc T, Cekmez F, Cetinkaya M, Kalayci T, Fidanci K (2015). Endocan and Soluble Triggering Receptor Expressed on Myeloid Cells-1 as Novel Markers for Neonatal Sepsis. Pediatr Neonatol.

[30] Fei Y, Wang W, Kwiecinski J, Josefsson E, Pullerits R, Jonsson IM (2011). The Combination of a Tumor Necrosis Factor Inhibitor and Antibiotic Alleviates Staphylococcal Arthritis and Sepsis in Mice. J Infect Dis.

[31] Riegels-Nielsen P, Frimodt-Moller N, Sorensen M, Jensen JS (1989). Antibiotic treatment insufficient for established septic arthritis. Staphylococcus aureus experiments in rabbits. Acta Orthop Scand.

[32] Ilhan N, Susam S, Gul HF, Bardas R, Ilhan N (2017). Which one is more effective for the treatment of rat sepsis model: thalidomide or etanercept?. Bratisl Lek Listy.

[33] Ilhan N, Susam S, Gül HF, Ilhan N (2019). The therapeutic effects of thalidomide and etanercept on septic rats exposed to lipopolysaccharide. Ulus Travma Acil Cerrahi Derg.

